# Ablation of typical atrial flutter as therapeutic component in carcinoid heart disease: a case report

**DOI:** 10.1186/s13256-022-03251-8

**Published:** 2022-02-02

**Authors:** Susann Groschke, Rolf Weinert, Björn Becker, Gert Richardt, Ralph Tölg, Leon Iden, Martin Borlich

**Affiliations:** Heart Center, Segeberger Kliniken GmbH (Academic Teaching Hospital of the Universities of Kiel, Lübeck and Hamburg), Am Kurpark 1, 23795 Bad Segeberg, Schleswig-Holstein Germany

**Keywords:** Carcinoid heart disease, Hedinger syndrome, Ablation, Typical atrial flutter, Neuroendocrine tumor

## Abstract

**Background:**

Carcinoid heart disease is the cardiac manifestation of carcinoid syndrome. There is limited research on rhythm management in patients with carcinoid heart disease. The association of typical atrial flutter and carcinoid heart disease in particular is poorly described.

**Case presentation:**

Here we present a case of a 77-year-old German woman with carcinoid heart disease and recurrent typical atrial flutter complicating the postoperative course after tricuspid valve replacement and its successful long-term rhythm control by ablation therapy.

**Conclusion:**

There is limited evidence on rhythm management in patients with the rare diagnosis of carcinoid heart disease. Typical atrial flutter repeatedly complicated the postoperative course of our patient with carcinoid heart disease and could finally be treated curatively by ablation. Radiofrequency ablation should be considered as a valuable therapeutic component in the challenging therapy of this disease.

## Background

Carcinoid heart disease (CHD) is a rare and challenging organ manifestation of neuroendocrine tumors (NET) caused by fibrotic remodeling processes, especially of the right ventricular endocardium, and is associated with a poor prognosis. Differentiated NET, together with poorly differentiated neuroendocrine carcinomas (NEC), belongs to the group of neuroendocrine neoplasms (NEN). Although well-differentiated, low-proliferative NET may be found incidentally in pathology specimens, some hormone-active NET lead to physical discomfort caused by the release of bioactive peptides. Carcinoid syndrome is the most common functionally active NET, in which tumor cells release large amounts of serotonin, tachykinins and transforming growth factor beta (TGF-ß), resulting in flush, diarrhea, and abdominal pain. Carcinoid tumors are predominantly found in the gastrointestinal tract, the bronchial system, and the urogenital tract [1]. In the case of progressive tumor growth with liver metastases and increased release of vasoactive substances, the hepatic first-pass effect gets lost, and the heart is exposed to increasing hormone concentrations that lead to the development of carcinoid heart disease, also known as Hedinger syndrome. The increasing hormone concentrations inside the heart lead to the deposition of fibrous plaques on the endocardium of the right-sided heart valves and ventricles, which can result in right heart failure that is difficult to treat. Little is known about the occurrence of arrhythmias in patients with CHD. Besides rarely occurring atrial fibrillation and ventricular tachycardia, typical atrial flutter has been reported only once as the initial symptom of carcinoid syndrome and CHD. We describe a case in which typical atrial flutter repeatedly complicated the postoperative course of a patient with CHD and could be treated definitively by ablation.

## Case presentation

**Fig. 1:**
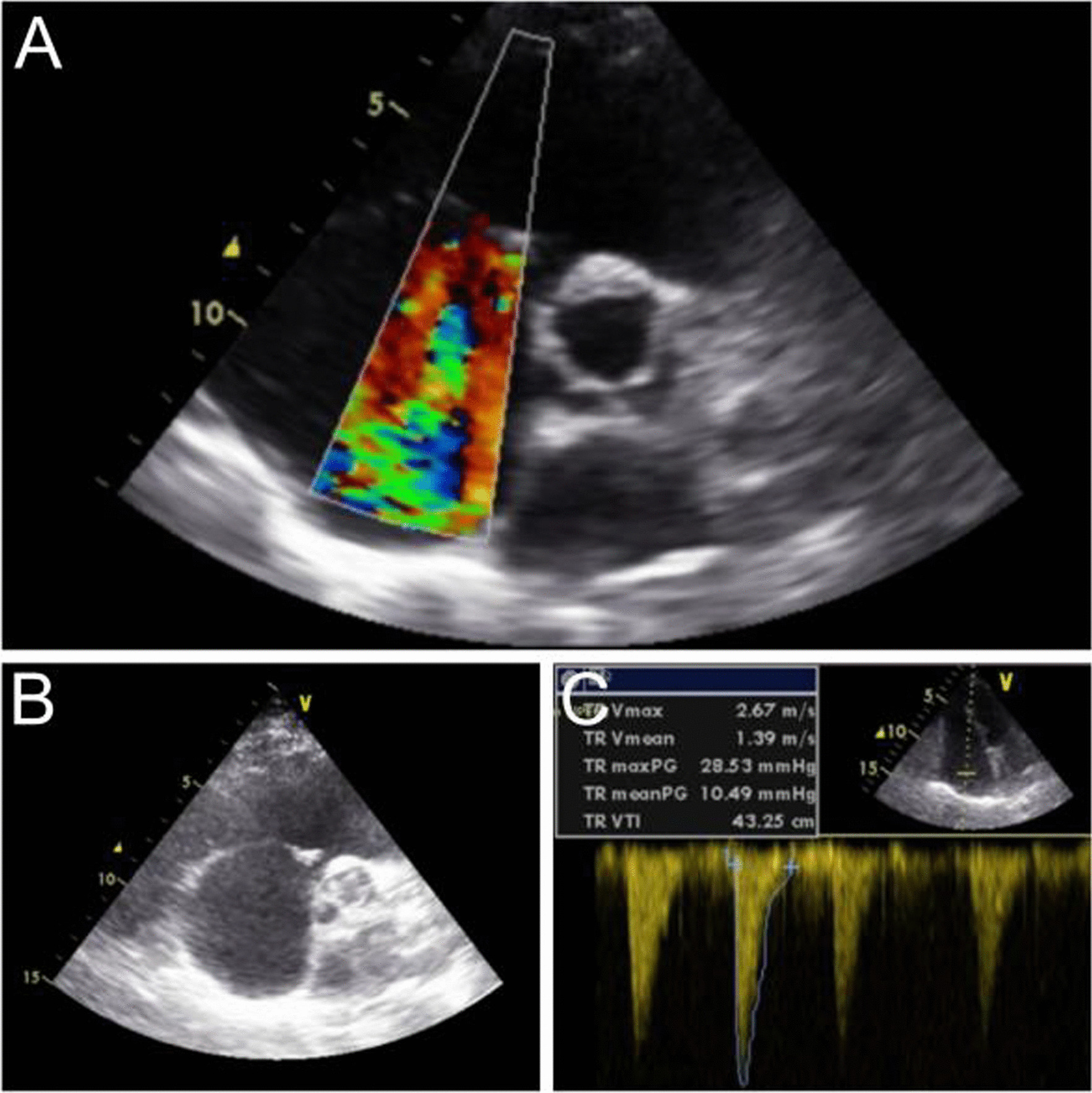
**A** Transthoracic echocardiogram (TTE) in parasternal short axis view with evidence of severe tricuspid regurgitation. **B** TTE in parasternal short axis view shows a dilated right heart. **C** Continuous wave spectral Doppler analysis of the tricuspid regurgitation (TR) jet for assessing pulmonary arterial pressure

A 77-year-old German woman presented to our emergency department with dyspnea and peripheral edema, which was first noted a few days ago. Previous ultrasound had ruled out deep vein thrombosis (DVT), and diuretic therapy had already been initiated leading to a slight alleviation of her symptoms. The medical history included tonsillectomy, appendectomy, and hysterectomy. No other previous diseases were known at that time. On physical examination, we noted a marked jugular vein pulse and discrete edema of the lower extremities. Pulmonary and cardiac auscultation revealed no abnormal findings. Electrocardiography (ECG) showed sinus rhythm with a heart rate of 95 beats per minute without further abnormalities. On transthoracic echocardiography we noticed a dilated right heart with severe tricuspid regurgitation (Fig. 1). No other cardiac defects were seen; the left ventricular systolic function was normal. Using continuous wave (CW) Doppler analysis for estimating the systolic pulmonary arterial pressure (sPAP), a value of 28 mmHg was calculated.

CT angiography subsequently ruled out pulmonary artery embolism. This examination also revealed multiple liver lesions, which we further investigated. Coronary angiography and right heart catheterization were performed first. There was no relevant coronary artery disease, no shunt defect, and slightly increased pulmonary artery pressure. Further internal and oncological diagnostics initially failed to detect a primary tumor as cause of the liver lesions. Therefore, a biopsy was performed with histological evidence of a well-differentiated G1 NET with high expression of synaptophysin and low co-expression of CD-56. The proliferation rate was 1%, validated with a MIB-1 marker.

**Fig. 2:**
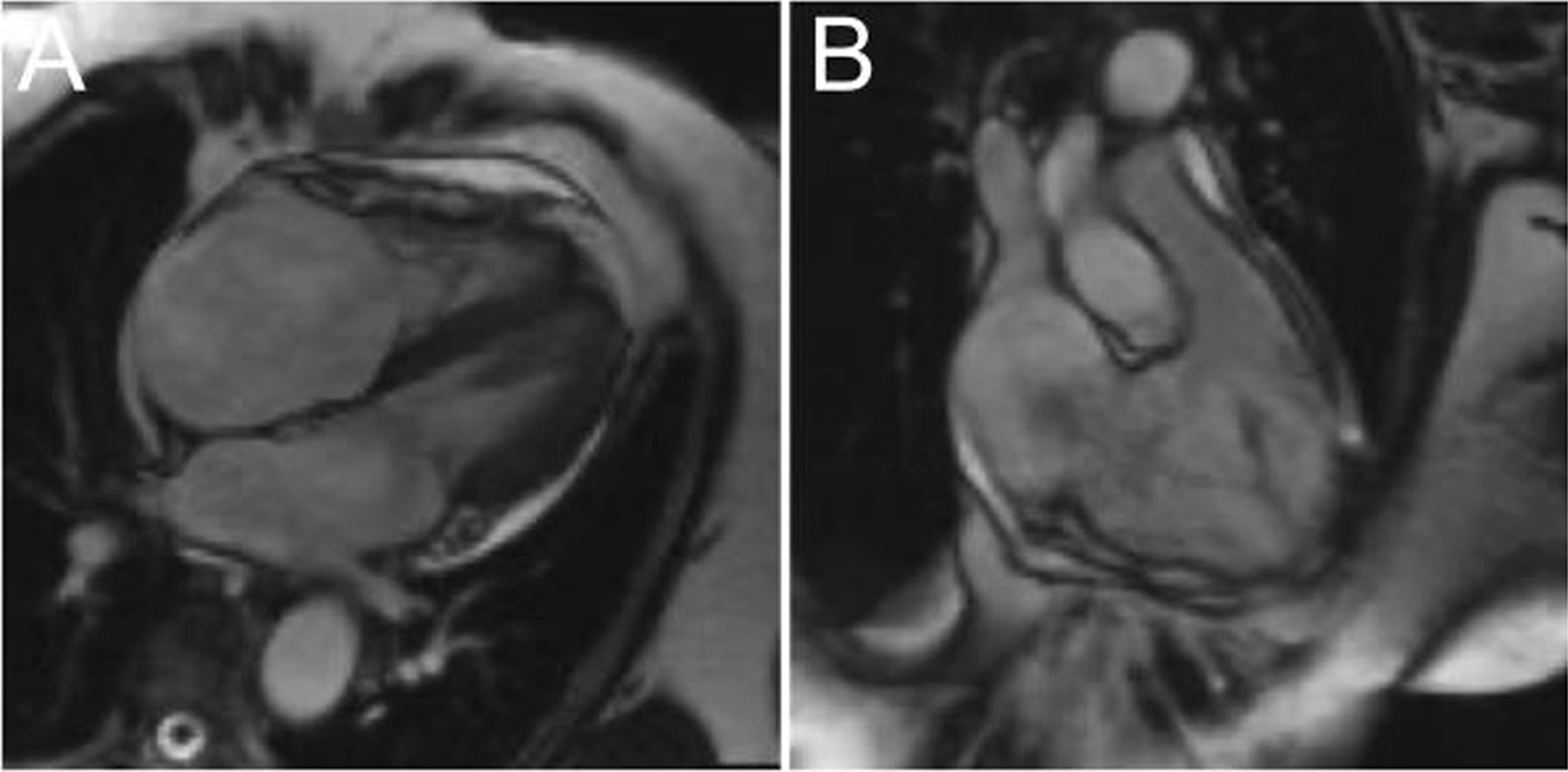
**A** MRI scan in four-chamber-view showing a highly dilated right atrium and a hypertrabeculized right ventricle. **B** MRI scan of the right ventricular outflow tract demonstrating the severe tricuspid regurgitation

In a 24-hour urine collection, the concentrations of 5-hydroxyindoleacetic acid and serotonin were both significantly elevated (72 mg/24 hours at an upper limit of 8 mg/24 hours and 277 µg/24 hours at an upper limit of 240 µg/24 hours, respectively). Serotonin levels in serum analysis were normal; however, preceding intake of pantoprazole may result in lowered serum serotonin levels. We assessed the findings as indicative of carcinoid syndrome and investigated cardiac involvement on magnetic resonance imaging (MRI), which showed severely dilated right heart cavities (Fig. 2). The right ventricular myocardial structure showed a hypertrabecularization. The tricuspid valve was rigid and fixed with incomplete closure. The other valves, especially the pulmonary valve, had no pathology. Cine Magnetic Resonance Imaging documented a severe systolic jet from the right ventricle into the right atrium caused by incomplete closure of the tricuspid valve.

The case was discussed interdisciplinarily with cardiologists, cardiothoracic surgeons, and oncologists. The tumor entity was judged to be associated with a good prognosis owing to its slow proliferation rate. Drug treatment was initiated with intramuscular injection of somatostatin. The right heart pathology was deemed to be most relevant regarding symptoms and prognosis. After joint discussion, there was agreement on a protocol involving two octreotide cycles followed by mechanical tricuspid valve replacement. The liver metastases with diameters up to 5 cm were treated conservatively.

The decision for a mechanical heart valve was made due to the lower risk of fibrosis compared with biological valve replacement. The patient was compliant and agreed to long-term strict [international normalized ratio (INR) 3.0–3.5] oral anticoagulation with phenprocoumon. The operation was performed 5 months after the first hospitalization in June 2015. A 31 mm Carbomedics heart valve was implanted. The MRI and echo findings were confirmed* in situ*. The tricuspid valve was found to be rigid and retracted with no possibility of valve reconstruction. In the postoperative course, a hemopericardium occurred with the need for surgical treatment as well as the first onset of typical atrial flutter, which was successfully treated with amiodarone. The patient was discharged 16 days after the tricuspid valve replacement.

In October 2017, the patient presented with recurrent typical atrial flutter and dyspnea. Regarding carcinoid heart disease with an otherwise good postoperative course, we recommended ablation therapy owing to its good long-term efficacy [2]. Twelve-lead-ECG suggested a counterclockwise, cavotricuspid isthmus-dependent atrial flutter with 2:1 conduction and a ventricular rate of 130 beats per minute.

**Fig. 3:**
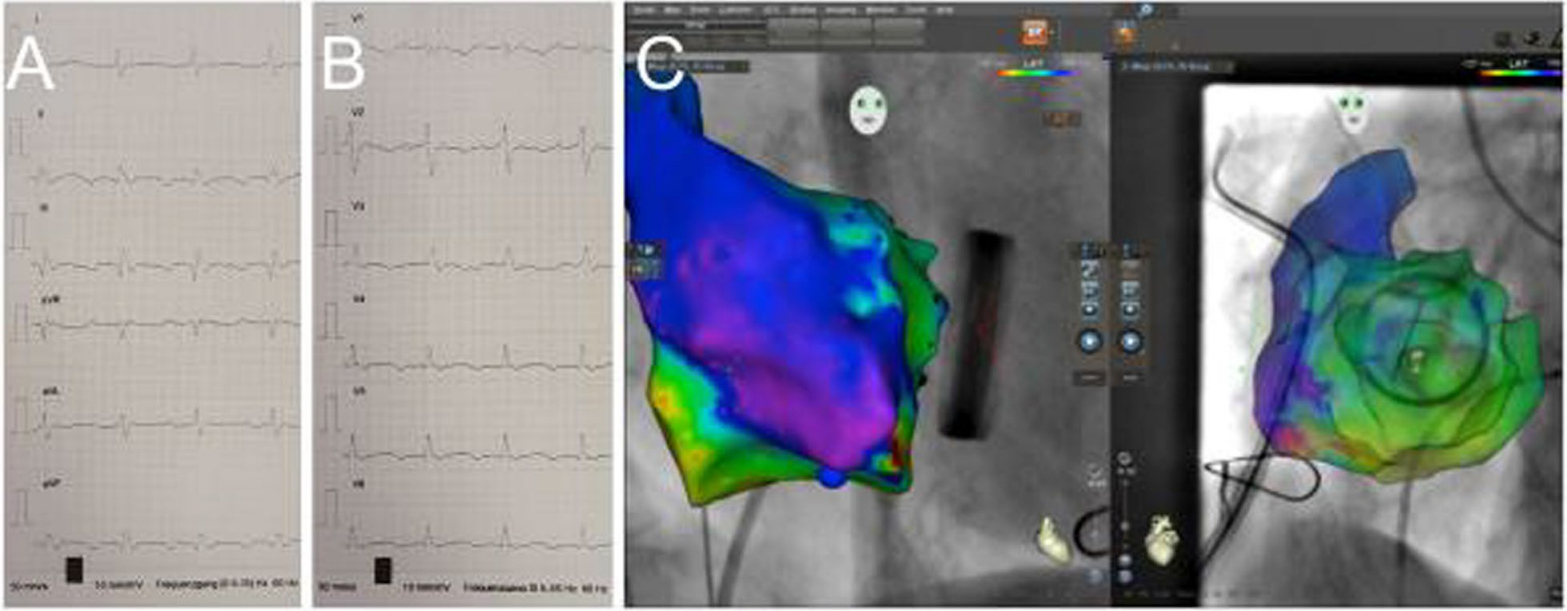
ECG during typical atrial flutter with limb leads (**A**) and chest leads (**B**). **C** Electroanatomic reconstruction of the right atrium with Local activation time (LAT) mapping visualizing the counterclockwise typical atrial flutter (CARTO 3, Biosense Webster). Fluoroscopic images are integrated into the 3D map using CARTO UNIVU. Black dots mark sites of metal artifact recordings initiated by contact of the ablation catheter with mechanical valve prosthesis (left: right anterior oblique (RAO) 30°, right: left anterior oblique (LAO) 45° view)

However, the presence of incisional or other types of scar-related atrial flutter was also considered. Invasively, the suspected tachycardia mechanism was confirmed by both entrainment and activation mapping (Fig. 3). After registration and superimposition of the fluoroscopy data, the position of the mechanical valve prosthesis was carefully marked by annotating the locations of the metal artifact recordings of the ablation catheter. Ablation was performed with an inferior cavotricuspid isthmus line leading to termination of the typical atrial flutter. The bidirectional block of the cavotricuspid isthmus was finally confirmed by differential pacing.

The patient completed a follow-up of 3 years after ablation and remains in good clinical condition. All available ECG recordings show sinus rhythm. No new arrhythmias have been noted. Concerning heart failure, she is asymptomatic [New York Heart Association (NYHA) I] under appropriate guideline-directed medication. As such, there is currently no evidence for a progression of the carcinoid heart disease.

## Discussion

Carcinoid heart disease (CHD) is a serious organ manifestation of patients with carcinoid syndrome and advanced neuroendocrine tumor stage, which lowers the 3-year survival rate to 31% [3, 4]. It was first described in 1954 [5]. Approximately 20–50% of patients with carcinoid syndrome are affected by CHD [6] also known as Hedinger syndrome. Characterized by severe right-sided valvular abnormalities, the disease is caused by enhanced fibrogenesis primarily triggered by serotonin (5-hydroxytryptamine) secreted by neuroendocrine tumors (NET). These NET typically arise in the gastrointestinal and genitourinary tracts. The incidence of carcinoid tumors is 1–2/100,000, with the vast majority of these tumors (> 90%) being benign [6]. Symptomatic CHD usually presents between 50 and 70 years of age [1]. Our patient, who was a 77-year-old lady at initial diagnosis, initially presented to us with signs of heart failure caused by severe tricuspid regurgitation, which is one of the major features of CHD patients. Diarrhea, skin flushing, or bronchospasm were not seen as typical symptoms of carcinoid syndrome in this case [6]. Because serotonin is not activated in the lungs, left-sided CHD is a rarity [1]. Arrhythmias are another very rare complicating feature of this disease; to date, only a single case of typical atrial flutter has been described as an initial manifestation of CHD [7]. Case reports of cardiac arrhythmias in CHD have been described predominantly in the context of carcinoid crisis, a life-threatening condition in which large amounts of serotonin and other vasoactive hormones are released during surgery or chemotherapy. Besides carcinoid crisis, few cases of atrial fibrillation, ventricular tachycardia, and premature ventricular contractions (PVC) have been published [8–10]. One study so far has demonstrated the feasibility of catheter ablation of atrial and ventricular tachyarrhythmias in a cohort of 17 patients with neuroendocrine tumors. Only two of these patients had carcinoid heart disease, and their treated arrhythmia has not been described [11].

In addition to right heart failure and valvular heart disease as causes of arrhythmia, serotonin can increase sympathetic activity and cardiac excitability, triggering these heart rhythm disorders [8]. In our case, typical atrial flutter occurred after successful surgical replacement of the tricuspid valve and complicated the postoperative course. After initial anti-arrhythmic therapy with amiodarone, the atrial flutter recurred. In the early postoperative period, triggers such as inflammation, fluid disturbances, and electrolyte shifts likely triggered the arrhythmia in addition to carcinoid heart disease with right atrial changes. When managed conservatively, typical atrial flutter has a high recurrence rate due to the anatomically fixed structures contributing to the reentrant circuit. Postoperative scarring as has been described for Ebstein anomaly after a tricuspid valve replacement did not lead to additional scar-related atypical right atrial flutter in this case, but possibly retriggered the typical isthmus-dependent atrial flutter by conduction delays. Radiofrequency ablation 2 years after the patient’s initial presentation at our clinic made it possible to achieve freedom from recurrence that persisted up to now. Our group recently published that early postoperative ablation of typical flutter in a mixed cardiosurgical cohort was associated with a favorable short- and long-term safety and efficacy profile and could be considered as part of the cardiac rhythm management options in this setting [12]. Early interventional therapy with a high success rate might thus have been an option for our patient in the early postoperative phase, as well.

With an overall poor prognosis of the disease, early identification of the underlying disease was crucial for our patient. The treatment focused on controlling the underlying carcinoid syndrome by initiation of treatment with somatostatin analog octreotide to decrease serotonin levels and consecutive surgical treatment of the severe tricuspid regurgitation [13]. Today, other drug therapy options are available for disease resistant to somatostatin, such as pasireotide or lutetium dotatate [14, 15]. We abstained from liver-directed therapies owing to the well-differentiated G1 NET of our patient with a low proliferation rate of 1% and the stable course of the disease after successful rhythm control.

The challenge in dealing with carcinoid heart patients is to provide a multidisciplinary and structured therapy, which was performed successfully in this case.

## Conclusion

Research on rhythm management in patients with carcinoid heart disease (CHD) is limited. Typical atrial flutter repeatedly complicated the postoperative course of our patient with CHD and could finally be treated curatively by ablation. RF ablation should be considered as a valuable therapeutic component in the challenging therapy of this disease.

## Data Availability

Case data derive from patient’s records and are therefore not publicly available.

## Abbreviations

CHD: Carcinoid heart disease
PVT: Deep vein thrombosis
NEC: Neuroendocrine carcinomas
NEN: Neuroendocrine neoplasms
NET: Neuroendocrine tumors
NYHA: New York Heart Association
PVC: Premature ventricular contractions
sPAP: Systolic pulmonary arterial pressure
